# An analysis of the energetic reward offered by field bean (*Vicia faba*) flowers: Nectar, pollen, and operative force

**DOI:** 10.1002/ece3.3851

**Published:** 2018-02-18

**Authors:** Emily J. Bailes, Jonathan G. Pattrick, Beverley J. Glover

**Affiliations:** ^1^ School of Biological Sciences Royal Holloway University of London Egham UK; ^2^ Department of Plant Sciences University of Cambridge Cambridge UK; ^3^ National Institute of Agricultural Botany Cambridge UK; ^4^ Department of Zoology University of Cambridge Cambridge UK

**Keywords:** *Bombus terrestris*, broad bean, bumblebee, faba bean, opening force, operative strength, plant breeding, pollination, sugar concentration preference

## Abstract

Global consumption of crops with a yield that is dependent on animal pollinators is growing, with greater areas planted each year. However, the floral traits that influence pollinator visitation are not usually the focus of breeding programmes, and therefore, it is likely that yield improvements may be made by optimizing floral traits to enhance pollinator visitation rates. We investigated the variation present in the floral reward of the bee‐pollinated crop *Vicia faba* (field bean). We examined the genetic potential for breeding flowers with a greater reward into current commercial varieties and used bee behavioral experiments to gain insight into the optimal nectar concentration to maximize bee preference. There was a large range of variation in the amount of pollen and nectar reward of flowers in the genotypes investigated. Bee behavioral experiments using nectar sugar concentrations found in *V. faba* lines suggest that *Bombus terrestris* prefers 55% w/w sugar solution over 40% w/w, but has no preference between 55% w/w and 68% w/w sugar solution. We provide a first indication of the force required to open *V. faba* flowers. Our results provide a valuable starting point toward breeding for varieties with optimized floral reward. Field studies are now needed to verify whether the genetic potential for breeding more rewarding flowers can translate into higher yield and yield stability.

## 1 INTRODUCTION

Pollinators are increasingly being recognized as important for global food security. Crops that produce higher yields when pollinated by animals represent around three‐quarters of the 115 most important crops (by tonnes produced; Klein et al., 2007). Furthermore, pollinator density has recently been reported to be the most important predictor of crop yield across many different crop systems, particularly in small farms (Garibaldi et al., 2016). The quality and therefore economic value of many crops are also known to be improved after animal pollination (eg. Garratt, Breeze et al., 2014; Klatt et al., 2014). Our demand for these pollinator‐dependent crops is growing globally (Aizen & Harder, 2009), and these yields are predicted to become increasingly more reliant on pollinators in the face of heat stress induced by climate change (Bishop, Jones, Lukac, & Potts, 2016).

Flowers are the interface at which a plant and pollinator interact, and their structure, color, scent, and reward, among other traits, will influence how likely an animal is to visit the flower. However, despite the reliance of crop yield on pollinators, breeding programmes do not generally select directly for floral traits, instead focusing on agronomic traits such as harvest index, drought resistance, and disease resistance (Kobayashi, Tsukamoto, Tanaka, Niikura, & Ohsawa, 2010; Richards, 2000; Tester & Langridge, 2010). Therefore, optimal floral trait combinations to attract pollinators and maintain high pollination rates may have been lost by genetic drift or selective sweeps. This has led to the suggestion that breeding crops through selecting for floral traits could lead to improvements in food security by attracting greater numbers of pollinators, as well as improving foraging resources for wild pollinator communities (e.g., Bailes, Ollerton, Pattrick, & Glover, 2015; Carruthers et al., 2017; Mallinger & Prasifka, 2017; Palmer, Perez, Ortiz‐Perez, Maalouf, & Suso, 2009).

A wealth of previous studies have shown that, where it is possible to differentiate between flowers, bees prefer flowers with larger rewards, usually in the form of pollen or nectar (Brunet, Thairu, Henss, Link, & Kluever, 2015; Cnaani, Thomson, & Papaj, 2006; Mallinger & Prasifka, 2017; Robertson, Mountjoy, Faulkner, Roberts, & Macnair, 1999; Whitney, Dyer, Chittka, Rands, & Glover, 2008). However, a flower with a large reward may not always maximize its pollination rate. An important point to consider is the accessibility of the reward. For example, the flowers of many members of the legume family (Fabaceae) require pollinators to apply force to access their reward. In some species, such as *Spartium junceum*, the force required is considerable and exceeds the strength of the honeybee *Apis mellifera* (Córdoba, Benitez‐Vieyra, & Cocucci, 2015; Córdoba & Cocucci, 2011). For accessible rewards, flowers with larger quantities (e.g. nectar volumes) will take longer to visit (Cresswell, 1999; Ollerton, Killick, Lamborn, Watts, & Whiston, 2007), which will reduce the number of flowers visited by an individual in a given amount of time. Furthermore, from a pollinator's perspective, intermediate sugar concentrations may be the most preferable. As the energy (sugar) content of nectar increases, so does its viscosity. As more viscous nectar takes longer to consume, above a certain concentration the extra energetic value of the solution will be offset by the time taken to consume it (Harder, 1986). Thus, multiple facets of the reward of a flower need to be considered when determining the most successful strategy to maximize pollination rates.

**Figure 1:**
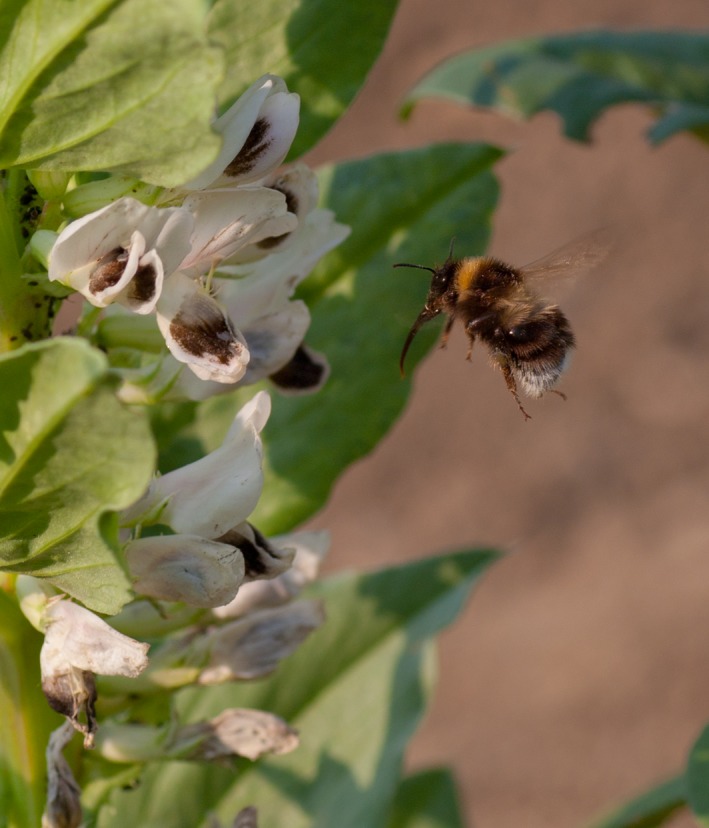
A bumblebee visiting a field bean (*V. faba*) flower

A good example of a crop where breeding for optimized floral traits could be highly beneficial is the field bean (*Vicia faba* L.; Figure 1). This crop is an important legume species, with over 2 million hectares grown worldwide in 2014, for both animal and human consumption (FAOSTAT, 2016). However, a major deterrent to farmers contemplating growing *V. faba* is its yield instability. Previous studies have shown that increases in bee visitation decrease yield variability in *V. faba* (Bishop et al., 2016; Cunningham & Le Feuvre, 2013), suggesting that yield instability may be linked to insufficient pollination. Furthermore, multiple studies have shown that bee pollination greatly enhances the yield of *V. faba*, with some reporting yield increases of over 50% compared with plants grown in the absence of pollinators (Bishop et al., 2016; Cunningham & Le Feuvre, 2013; Garratt, Coston et al., 2014; Nayak et al., 2015).

Within *V. faba,* there have been several studies of pollination and floral traits. Suso and colleagues have published multiple studies on how floral traits affect outcrossing (a proxy for bee visitation rate). They report that traits such as flag‐petal dimensions, number of inflorescences, and ovary length are associated with changes in outcrossing in populations of mixed genotypes (Suso & del Río, 2015; Suso, Harder, Moreno, & Maalouf, 2005; Suso & Maalouf, 2010; Suso, Nadal, Roman, & Gilsanz, 2008). Previous studies have also investigated the amount of pollen and nectar produced in selected lines of *V. faba* (Carré, Badenhauser, Taséi, Le Guen, & Mesquida, 1994; Kambal, Bond, & Toynbee‐Clarke, 1976; Osborne, Awmack, Clark, Williams, & Mills, 1997; Pierre et al., 1996; Suso et al., 2008). However, these studies examine only small panels of genotypes and very few have reported the quantity of nectar produced by genetically distinct lines. These studies of a limited number of genotypes may not be sufficient to identify the full range of variation available to breeders to select from. Furthermore, the relationship between the amount of pollen and nectar produced by flowers of *V. faba* has not yet been investigated. This information is important in establishing whether these traits can be bred for independently to develop an optimal reward combination to maximize pollination in this crop.

The aim of this study was to make a comprehensive assessment of the reward of *V. faba* flowers, focusing on the amount of nectar and pollen produced by a large number of distinct genotypes. The use of distinct genotypes was key to identifying heritable variation in the reward of flowers that could be selected for by breeders. We tested the hypothesis that there is a trade‐off in resource use in flowers, such that the amount of nectar and pollen produced are negatively correlated and cannot be targeted independently by breeding programmes. Furthermore, for the first time, we quantify the force required for a bee to open a flower of *V. faba* and access its reward. Finally, using behavioral experiments under controlled conditions, we explored bumblebee preference for *V. faba* relevant sugar concentrations, since previous studies have focused on nectar volume or lower sugar concentrations (e.g., Cnaani et al., 2006; Waddington & Gottlieb, 1990). The data we report provide novel insight into the range of pollinator reward present in a single species, while suggesting strategies for breeding crop varieties with enhanced visitation rates and benefit for pollinators.

## 2 MATERIALS AND METHODS

### 2.1 Study species and growth conditions

We analyzed 30 lines of *V. faba* from the National Institute of Agricultural Botany (Cambridge, UK) seed collection (Table S1). These lines were selected randomly with regard to the reward that they produced. All lines had been self‐pollinated for a minimum of five generations (with the exception of NV706 which was in generations 0–2 of self‐pollination) to minimize genetic variation within a line. Plants were grown in an insect‐proof temperature‐controlled glasshouse between September and May of 2012–2015, to minimize environmental variation between months. As it was not possible to grow all plants simultaneously due to time and space constraints, plants within a line were grown across several different months to control for differences in the growing conditions. Glasshouse conditions were maintained at 18–25°C with 16–18 hr daylight, depending on the month. When daylight levels fell below 20,000 lux, 10,000 lux high‐pressure sodium lights were automatically activated.

### 2.2 Measurement of floral traits

The number of pollen grains produced by a flower was calculated by resuspending pollen grains from ten flowers of a single plant together in a Tween80‐agar solution then counting samples under the microscope following the method of Kambal et al. (1976; see Supporting Information). Sample size ranged from 5 to 8 plants (median = 6; Table 1). Months that lines were measured in are given in Figure S1. Pollen production was not scored for lines NV175 and NV574.

**Table 1:** The level of replication for models of the nectar properties and pollen production of lines. Pollen production was estimated from a pooled sample of flowers. For n flowers/plant the number of flowers on each plant is separated by commas, which each number representing one plant replicate. Analyses of the mass of sugar produced by a flower were calculated ^1^including estimated data for low volumes where a true reading of sugar concentration was not available, but excluding lines NV155 & NV658 (main text) or ^2^with only flowers for which sugar concentration could directly measured

Line		NV020	NV027	NV079	NV082	NV100	NV129	NV155	NV175	NV293	NV490	NV574	NV604	NV619	NV620	NV626	NV639	NV640	NV641	NV643	NV644	NV648	NV649	NV650	NV653	NV658	NV671	NV673	NV675	NV676	NV706
n plants	Pollen	6	6	6	8	6	5	5	‐	5	6	‐	6	6	5	5	8	7	8	5	7	6	6	7	6	6	5	5	5	7	6
	Volume^a^	6	5	8	6	8	6	9/‐	4	8	7	6	7	10	6	6	13	7	21	9	7	8	5	7	6	11/‐	5	7	6	6	8
	Conc^b^	6	5	7	6	7	6	‐	4	8	6	6	7	10	6	6	13	7	21	9	6	7	5	7	4	‐	5	7	6	6	8
n flowers/plant	Volume^a^ analyses	5, 8, 9, 10, 10, 11	8, 9, 9, 9, 10	5, 5, 5, 7, 8, 9, 9, 9	6, 8, 8, 11, 11, 11	5, 5, 6, 8, 9, 10, 10, 12	7, 9, 9, 9, 9, 12	5, 5, 5, 8, 8, 9, 9, 9, 10	8, 8, 9, 9	5, 5, 8, 9, 9, 9, 10, 11	5, 9, 9, 10, 10, 10, 12	6, 8, 9, 9, 9, 10	7, 8, 9, 9, 9, 10, 11	5, 9, 10, 10, 10, 10, 10, 10, 11, 11	9, 9, 9, 9, 9, 15	9, 9, 9, 9, 9, 10	5, 6, 6, 6, 7, 10, 10, 10, 10, 10, 11, 11, 11	9, 9, 9, 10, 10, 10, 12	5, 5, 6, 7, 7, 7, 10, 10, 10, 10, 10, 10, 10, 11, 11, 12, 13, 13, 13, 15, 17	5, 5, 6, 8, 9, 9, 10, 11, 12	10, 10, 10, 11, 11, 12, 12	5, 6, 6, 8, 9, 9, 9, 9	5, 6, 9, 10, 12	6, 9, 9, 10, 10, 11, 12	5, 7, 9, 9, 9, 10	5, 5, 5, 6, 6, 6, 9, 9, 9, 9, 10	9, 9, 9, 9, 10	6, 8, 8, 9, 9, 10, 12	7, 9, 9, 10, 10, 11	8, 9, 9, 9, 10, 10	5, 8, 9, 9, 9, 9, 9, 10
	Sugar concentration^b^ analyses	4, 5, 9, 9, 9, 10	5, 8, 9, 9, 10	2, 3, 6, 7, 8, 9, 9	6, 7, 7, 10, 10, 11	2, 3, 5, 6, 7, 8, 10	2, 2, 3, 3, 5, 6	‐	8, 8, 9, 9	9, 4, 5, 8, 8, 8, 9, 9, 10	2, 7, 8, 8, 10, 10	6, 8, 9, 9, 9, 10	1, 1, 2, 3, 3, 5, 7	5, 9, 10, 10, 10, 10, 10, 10, 11, 11	1, 2, 2, 3, 3, 5	8, 9, 9, 9, 9, 9	1, 2, 4, 5, 9, 9, 9, 10, 10, 10, 10, 10	9, 9, 10, 10, 10, 10	3, 4, 5, 5, 5, 7, 7, 7, 10, 10, 10, 10, 10, 10, 10, 11, 11, 13,13, 14, 17	1, 4, 5, 6, 8, 9, 9, 10, 11	5, 5, 7, 8, 10, 11	2, 4, 5, 5, 5, 6, 7	4, 5, 6, 7, 10	6, 8, 8, 9, 9, 10, 12	2, 4, 6, 6	‐	9, 9, 9, 9, 10	6, 7, 7, 9, 9, 10, 11	7, 8, 9, 9, 10, 10	8, 8, 9, 9, 9, 10	2, 4, 4, 6, 6, 8, 8, 10

To estimate the nectar production of flowers, open flowers of stage four to five, as described by Osborne et al. (1997), were removed between 10 a.m. and noon from plants that had been flowering between 1 and 3 weeks in a random order within a day. The nectar was then extracted by centrifugation and the volume of nectar produced estimated based on the weight of the nectar collected (see Supporting Information).

The concentration of nectar collected by centrifugation was determined using one of two handheld refractometers (Bellingham + Stanley, Eclipse 45‐03 and Bellingham + Stanley, Eclipse 45‐82). The mass of sugar (mg) per flower was calculated from the sugar concentration and nectar weight (Supporting Information).

Nectar properties were quantified for a minimum of five flowers per plant (median = 9) for 4–21 plants per line (median = 7; Table 1), for each of 30 lines. Line NV641 was measured throughout the study period, months other lines were grown are indicated in Figures S2 and S3. Data for each individual flower were included in statistical analyses.

### 2.3 Measurement of floral traits: operative strength of a flower

The operative strength of a flower (equivalent to the force a pollinator needs to exert to trip a flower) was measured for two lines chosen for their different flower sizes (NV641 and the smaller flowered NV155). This was performed on open flowers using a method adjusted from Córdoba and Cocucci (2011) (see Supporting Information; Figure S4). Measurements were made between 11.30 a.m. and 1 p.m. Flowers were measured from six and five plants for lines NV641 (10, 12, 12, 10, 10, and 10 flowers) and NV155 (5, 11, 11, 10, and 9 flowers), respectively.

### 2.4 Bee behavioral experiments

To determine whether bumblebee foragers [*Bombus terrestris audax* (supplied by Agralan, UK)] have a preference between different sugar concentrations, pair‐wise comparisons were made, using the combinations 40% w/w and 55% w/w sugar solution, and 55% w/w and 68% w/w sugar solution. These were chosen to represent the mean sugar concentration of nectar across our variation panel, the highest mean sugar concentration of nectar within a line, and the highest plant mean sugar concentration in our dataset, respectively. Experiments were carried out in a 0.3 × 0.75 × 1.12 m plywood flight arena with a clear UV‐transparent Pexiglass lid. Before the experiment, colonies were fed *ad libitum* with ~30% w/w sugar solution and pollen. Bees cannot determine sugar concentration without making contact with the solution. Therefore, for each pair‐wise comparison, each sugar concentration was paired with a yellow or white colored disk, containing 5 μl sugar solution. This color cue allowed us to assess the forager's sugar concentration preference once they had learnt to associate the two “flower” traits. Following a training foraging bout (Supporting Information), 100 sequential choices (feeding) of an individual forager were recorded. As a forager depleted the reward of each disk, it was replaced with a fresh disk with sugar reward in a new location in the arena. The choices of 10 foragers were recorded. To control for color preferences, five foragers were assigned the high sugar concentration paired with white disks, and five foragers were assigned the high sugar concentration paired with yellow disks.

### 2.5 Statistical analyses

#### 2.5.1 Nectar analyses

We modeled how nectar properties varied between lines using linear mixed models (LMM). To account for uneven sample sizes between lines, models were fit using maximum likelihood in the nlme package (Pinheiro, Bates, DebRoy, Sarkar & R Core Team, 2017) in R version 3.4.1 (R Core Team, 2017). All models included the categorical explanatory variables Line (the plant “genotype”) and Month (month the measurement was taken in). To account for pseudoreplication from measuring each plant multiple times, Plant was included as a random effect (coded so that plants were nested within lines). Equality of variances and normality of errors were examined using residual plots. NectarVolume (volume of nectar produced per flower in μl) and SugarMass (total sugar produced per flower in mg) were transformed to Ln(NectarVolume + 0.28) and √(SugarMass + 0.1) to meet the model assumptions. SugarConcentration (% w/w sugar concentration of nectar) was not transformed. Lines NV155 and NV639 were excluded from sugar mass and concentration analyses because of the consistently low volumes of nectar they produced, which precluded accurate measurement of sugar concentration (Supporting Information). For SugarMass, we report analyses including estimated data for flowers with low nectar volume, where sugar concentration measurements were not possible. This reduced any potential bias in predicted line means from not including these flowers. The removal of these flowers from the dataset does change the means for some lines; however, it does not produce large qualitative differences in the results (see Supporting Information results 1). The effect of Line and Month on the respective nectar traits was tested using likelihood ratio tests of nested models. Line estimates given in the text were calculated using sum contrasts, 95% confidence intervals were calculated as ±1.96 × *SE*.

#### 2.5.2 Pollen analyses

Analysis of the pollen production (number of grains produced per flower) of lines was carried out using least squares (LS) regression models in JMP 11.0. The initial model included the categorical explanatory variables Line and Month. Month was removed from the final model due to its non‐significant effect. The relationship between the pollen and nectar production of lines was investigated by calculating Pearson's product‐moment correlation in R version 3.0.2. For each line, the estimated means from the models above were used, for sugar content and volume means, the transformed means were retained to satisfy the assumptions of homoscedasticity and linearity. A *t* test was used to test if the correlation coefficient was significantly different from zero.

#### 2.5.3 Force analyses

Analysis of the flower operative strength (mN) between lines was carried out using linear mixed models (LMM), fit using maximum likelihood in the lme4 package (Bates, Mächler, Bolker, & Walker, 2015) of R version 3.4.1 (R Core Team, 2017). The model included the categorical explanatory factors Line, Date (the date on which the measurement was taken), and Plant (coded so that plants were nested within lines to account for pseudoreplication). Date and Plant were coded as random effects. The effect of Line on operative strength was tested using likelihood ratio tests of nested models. Equality of variances and normality of errors were examined using residual plots. Line estimates given in the text were calculated using sum contrasts, 95% confidence intervals were calculated as ±1.96 × *SE*.

#### 2.5.4 Bee preference analyses

To test for a preference between the pairs of sugar concentration, a logistic regression was run using R version 3.0.2 with 1 assigned to choices of the higher sugar concentration and 0 to the lower sugar concentration. This followed the method of Groen et al. (2016), with the exception that forager choices were not aggregated into 10 choice bins.

## 3 RESULTS

### 3.1 The pollen production of flowers

**Figure 2:**
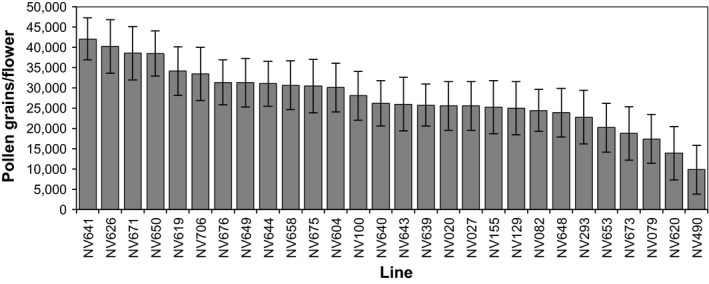
The average pollen production of *V. faba* lines (grains per flower). Data are means with 95% confidence intervals (*n* = 5–8). The pollen production of flowers is significantly different between lines (*F*_27,140 _= 6.23, *p* < .0001)

There was over a 4‐fold variation in pollen production measured between lines of *V. faba* (Figure 2). Pollen production (mean number of pollen grains per flower [95% CI]) ranged from 9,815 [3,794, 15,836] in NV490 to 42,083 [36,869, 47,298] in NV641, with a mean of 27,513. Month was not a significant predictor of pollen production (*F*_6,134 _= 1.35, *p* = .238) in the initial model PollenContent = Line + Month. In the final model PollenContent = Line, Line was a highly significant predictor of the amount of pollen produced per flower (*F*_27,140 _= 6.23, *p* < .0001).

### 3.2 The nectar production of flowers

**Figure 3:**
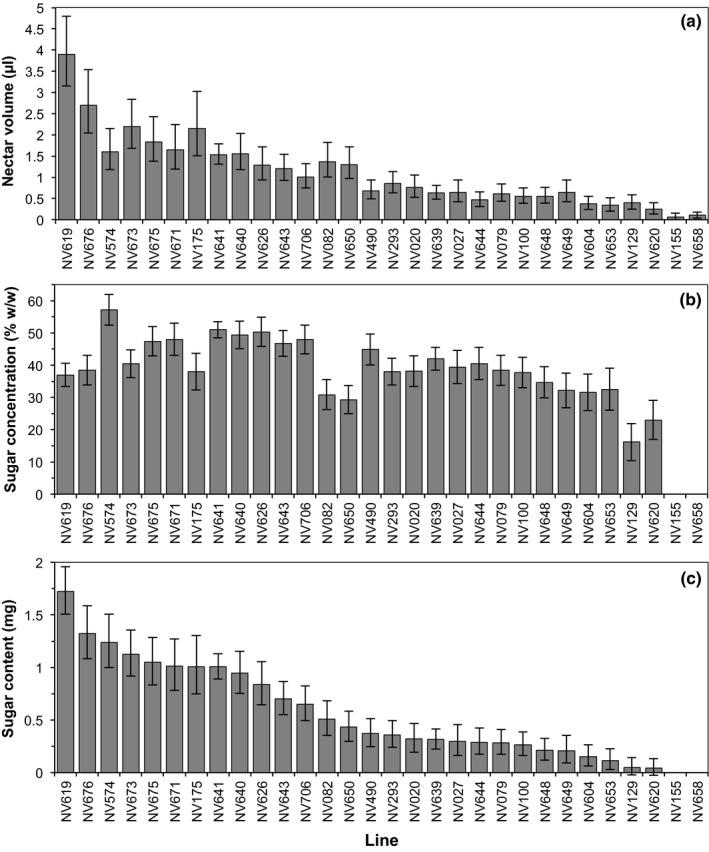
The nectar production of *V. faba* flowers between lines (predicted means with 95% confidence intervals). (a) The back‐transformed volume of nectar produced (μl) per flower. Line was a significant predictor of Ln(nectar volume + 0.28) (likelihood ratio = 361, *p* < .0001) (b) The sugar concentration of nectar (% w/w). Line was a significant predictor of nectar concentration (likelihood ratio = 219, *p* < .0001). (c) The back‐transformed total amount of sugar produced per flower (mg sucrose equivalents). Line was a significant predictor of √(sugar mass (mg) + 0.1) of a flower (likelihood ratio = 334, *p* < .0001). The concentration (% w/w) and sugar mass (mg) of nectar could not be determined for lines NV658 and NV155

The volume of nectar produced by flowers (back‐transformed mean [95% CI]) ranged from 0.1 μl [0.0, 0.1] in NV155 to 3.9 μl [3.5, 4.3] in NV619, with a mean of 1.1 μl (Figure 3a). Line (likelihood ratio = 361, *p* < .0001) and Month (likelihood ratio = 81, *p* < .0001) were significant predictors of the volume of nectar produced per flower. For the full model, Akaike information criterion (AIC) = 2,935, which increased to 3237 and 3004 when the factors Line and Month were removed, respectively.

More than a 3‐fold difference in nectar sugar concentration (% w/w) was observed between the 28 lines that consistently produced enough nectar to quantify sugar concentration. Sugar concentrations (% w/w) ranged from 16 [13, 19] in NV129 to 57 [55, 60] in NV574, with a mean of 39% w/w (Figure 3b). Line (likelihood ratio = 219, *p* < .0001) and Month (likelihood ratio = 90, *p* < .0001) were both significant predictors of nectar concentration. For the full model, AIC = 11162, which increased to 11327 and 11240 when the factors Line and Month were removed, respectively.

Overall, the total sugar (mg sucrose equivalents) produced per flower (Figure 3c) ranged from <0.1 mg/flower in lines NV620 (0.0 [0.0, 0.1]) and NV129 (0.0 [0.0, 0.1]) to a maximum of 1.7 [1.6, 1.8] mg/flower in line NV619 and mean of 0.6 mg/flower. Line (likelihood ratio = 334, *p* < .0001) and Month (likelihood ratio = 63, *p* < .0001) were both significant predictors of the sugar production of a flower. For the full model, AIC = −969, which increased to −689 and −918 when the factors Line and Month were removed, respectively.

### 3.3 The sugar concentration preferred by bumblebees

**Figure 4:**
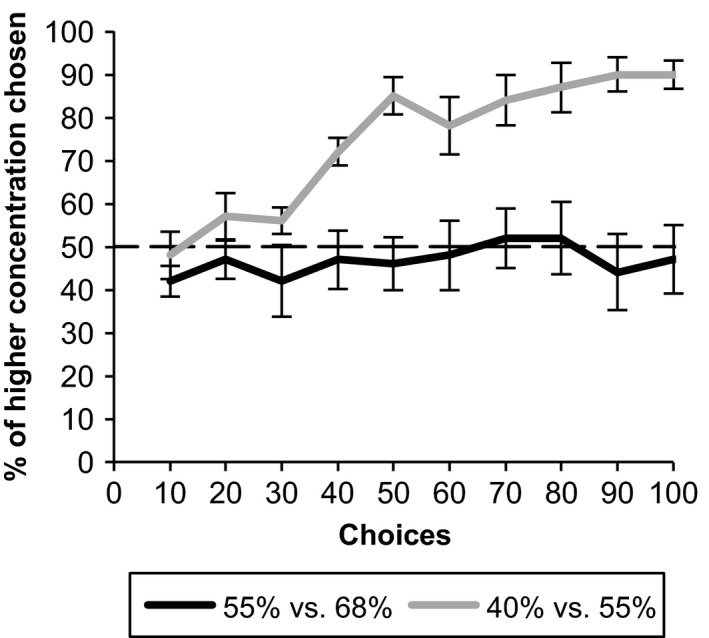
The preference of *B. terrestris* for *V. faba* relevant nectar sugar concentrations. The average preference for the higher concentration of sugar solution of ten foragers for 40% versus 55% sugar solution or 55% versus 68% w/w sugar solution as they learned to associate these rewards with color stimuli. Choices are averaged over 10 successive choices for each bee. There was a significant preference for 55% over 40% w/w sugar (χ^2^(1) = 132.53, *p* < .0001) but not between 68% and 55% w/w sugar (χ^2^(1) = 0.78, *p* = .38)

All bees showed a preference for 55% w/w sugar versus 40% w/w sugar solution after 100 choices (Figure 4), regardless of the color assignment of the two sugar concentrations. At 91–100 choices, the proportion of disks assigned to 55% w/w sugar visited was 90% ± 6 or 90% ± 3 when disks were colored yellow or white, respectively (mean ± *SE*). Over the course of the experiment, there was a significant increase in the proportion of higher concentration disks visited by foragers (χ^2^(1) = 132.53, *p* < .0001).

Conversely, individual bees showed no preference when offered 55% w/w sugar versus 68% w/w sugar solution (Figure 4). At 91–100 choices, the proportion of disks assigned to 68% w/w sugar chosen was 50% ± 11 or 44% ± 12 when disks were colored yellow or white, respectively. There was no significant change in the proportion of higher sugar concentration disks visited over the course of the experiment (χ^2^(1) = .78, *p* = .38).

### 3.4 The relationship between the nectar and pollen content of lines

**Figure 5:**
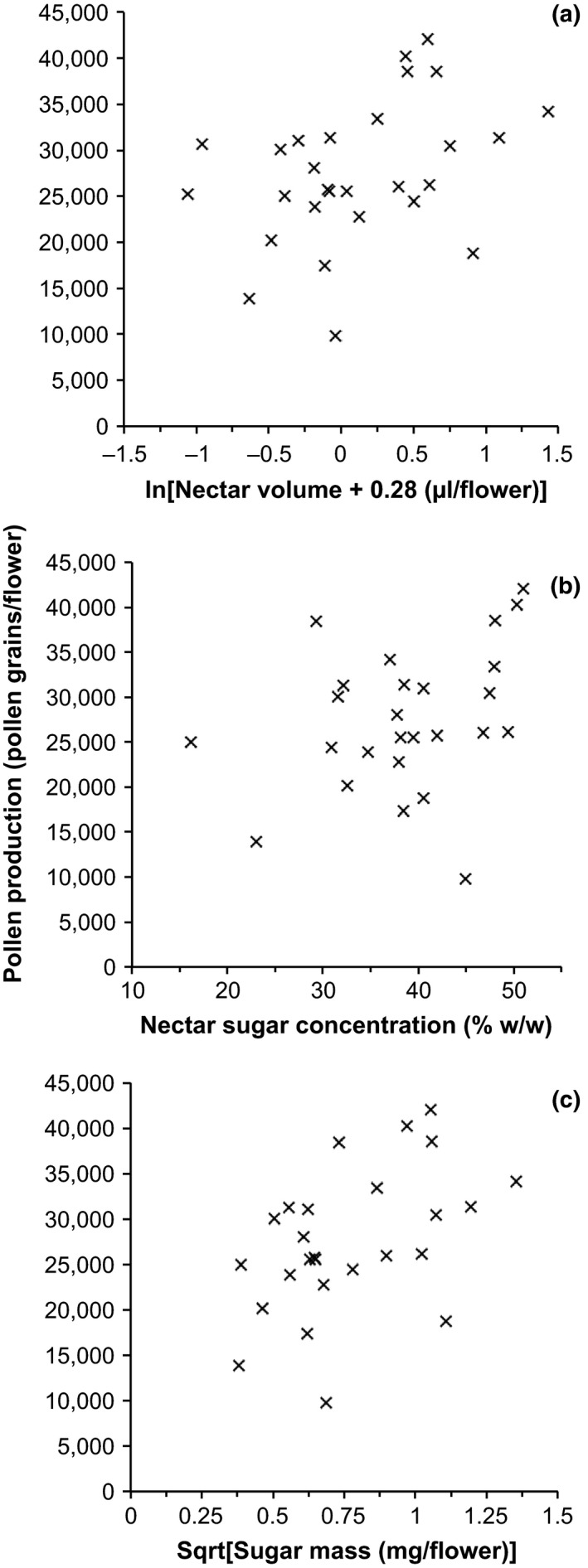
The relationship between pollen production (grains/flower) and various measures of nectar production in *V. faba* lines. Each point represents an individual *V. faba* line mean (from Figures 2 and 3). (a) The relationship with nectar volume produced by a flower (μl). Nectar volume is ln transformed (*n* = 28, *R* ^2 ^= .14, *t* = 2.04, *p* = .052). (b) The relationship with nectar sugar concentration (% w/w) (*n* = 26, *R* ^2 ^= .11, *t* = 2.62, *p* = .096). (c) The relationship with the sugar mass (mg) produced by a flower. Sugar mass is square root transformed (*n* = 26, *R* ^2 ^= .22, *t* = 2.61, *p* = .015)

There was a weak but significant positive relationship between the amount of pollen produced and the overall sugar content of the lines analyzed (*R* ^2 ^= .22, *t* = 2.61, *p* = .015). The relationships between pollen production and sugar concentration (*R* ^2 ^= .11, *t* = 2.62, *p* = .096), and pollen production and volume of nectar produced by flowers (*R* ^2 ^= .14, *t* = 2.04, *p* = .052), were also positive but only marginally significant (Figure 5).

### 3.5 The force required to open a flower

The force required to open flowers of lines NV155 and NV641 was estimated to be 17.1 [12.3, 21.9] and 20.1 mN [15.3, 24.8], respectively (predicted mean [95% CI]). These operative forces were significantly different between lines (likelihood ratio = 4.5, *p* = .035). For the full model, AIC = 702.7, which increased to 705.1 when Line was removed.

## 4 DISCUSSION

The potential to breed crops that have higher visitation rates and are more beneficial for pollinators depends on the presence of variation in relevant floral traits. Any variation present must also be genetically determined. In this study, we have demonstrated that there is substantial variation between genetically distinct lines in the amount of nectar and pollen rewards produced by flowers of the crop *V. faba*. This suggests that breeding for flowers with altered floral traits that enhance visitation rates and/or have greater capacity to support pollinator populations in *V. faba* is possible, although its value will depend on the result of future yield tests. While the month the plants were grown in had a significant effect on nectar (but not pollen) production, the plant genotype (Line) always improved model fit to a much greater extent (as seen from larger likelihood ratios and a greater reduction in AIC when Line compared with Month is added to our model), indicating that genetic variation was more important than environmental variation (under controlled growth conditions) in determining the overall sugar production of a flower.

The pollen production measured in our study is comparable to that seen in other studies of fewer lines (e.g., Carré et al., 1994; Suso et al., 2008) suggesting that *V. faba* lines may already be close to the upper limit for pollen production in this species. In contrast, for nectar production (a trait for which there is much less data in the literature), we identified several lines with substantially higher values than in previous reports by Pierre et al. (1996) and Osborne et al. (1997), in terms of total sugar produced per flower (up to ~4 times greater), sugar concentration (up to ~1.7 times greater), and volume produced per flower (over double).

Recently, it has been shown that more pollen analog is transferred to artificial flowers with a higher sugar concentration when bees are allowed to choose freely between two different concentrations (Thomson et al., 2012). This suggests that improving the sugar concentration of flowers will be a successful strategy to improve pollination rates in *V. faba*. Here, using controlled experiments with nectar‐realistic volumes in each flower, we have shown that bees prefer a sugar concentration of 55% w/w over 40% w/w. This result is in line with theoretical predictions that the concentration that maximizes a bee's energetic gain is 50%–60% (Harder, 1986). This optimum maximizes the trade‐off between the increased energy density of the solution and reduction in consumption rate with increasing solution concentration and viscosity. Interestingly, we found that bees have no preference between sugar solutions of 55% and 68% w/w. Our findings improve on previous work investigating bee preference for high sugar concentrations (e.g., Mommaerts, Wäckers, & Smagghe, 2013; Waller, 1972; Woodrow, 1968) using realistic volumes of reward within a flower under free flight conditions with a single forager present so that choices are not influenced by social cues. In combination with the findings of Thomson et al. (2012), the results of this study suggest that breeding for nectar sugar concentrations up to 55% w/w nectar will improve pollinator preference and pollen transfer. However, breeding beyond this concentration would have no further beneficial effects and not only waste plant resources but also increase the amount of time it takes for a bee to visit a flower (Harder, 1986).

With respect to nectar volume, it may not be advantageous for crop production to breed for plants with values higher than the average of our dataset, ~1 μl. The length of time spent on a flower foraging for nectar increases with the nectar volume (Cresswell, 1999; Ollerton et al., 2007). This increased visitation time has been shown to increase pollen deposition and export by pollinators in some systems (e.g., Ollerton et al., 2007). Nonetheless, it will also reduce the number of visits made to the crop in a given amount of time and the number of visits required to fill the bee's crop. Combined, these factors may lead to a higher rate of self‐pollination in flowers producing greater volumes of nectar and lower visitation rates by individual foragers. Indeed, Suso et al. (2005) reported a negative correlation between outcrossing and nectar volume in some of the synthetic populations they have examined. *V. faba* yield still benefits from flower visitation in a self‐pollination scenario (Kambal et al., 1976), and the reduced number of flower visits by a single pollinator will be counteracted by increased numbers of flower visitors, to some extent. For example, Pierre et al. (1996) found in a pair‐wise comparison that there was a higher visitation rate by bumblebees to the bean line that produced higher nectar volumes. However, outcrossing is beneficial to maintain yield stability during extreme environmental conditions and to maintain a high yield in future generations (Nadal, Suso, & Moreno, 2003). Therefore, breeding for a higher nectar concentration (rather than volume), up to a threshold value of 55% w/w, may be a more viable approach to improve bee preference for flowers while maintaining adequate pollination rates. Contrary to this, if the aim is to support pollinator populations, maximizing the volume of nectar and sugar content of flowers would be a feasible strategy.

While nectar is energetically a less costly reward for the plant to produce, pollen production may also be an important route for improving pollination rates. *B. terrestris* and other bee species will frequently rob or secondarily rob *V. faba* flowers of nectar rather than visiting them legitimately (Garratt, Coston et al., 2014; Emily J. Bailes personal observation). However, to obtain pollen from the flowers, bees must visit legitimately. Enhancing the quality and quantity of pollen produced may therefore be more effective at increasing legitimate visitation rates to the crop. It has been demonstrated that bees can determine the nutritional quality of pollen by taste and prefer pollen with high concentrations of essential amino acids and ratio of protein:lipids up until a threshold of ~10:1 (Cook, Awmack, Murray, & Williams, 2003; Somme et al., 2015; Vaudo, Patch, Mortensen, Tooker, & Grozinger, 2016). Furthermore, in *V. faba*, pollen production within the range of this study has been shown to be positively correlated with outcrossing in the field (Suso et al., 2008). This enhancement of pollen quantity and quality will come at the cost of extra resource use within the plant, particularly macronutrients such as nitrogen and phosphorus (Ashman, 1994). Further investigation is therefore needed to both determine whether the *quality* of pollen is genetically determined in *V. faba*, and whether producing large quantities of high‐quality pollen will impact the overall yield of the plant.

Should future studies demonstrate that enhancing the pollen reward of flowers is a viable strategy, this should be achieved while maintaining some nectar secretion. Bumblebees do not generally specialize on pollen or nectar collection, even within a single foraging bout (Hagbery and Nieh, 2012; Konzmann and Lunau, 2014). Therefore, to improve the likelihood of an individual returning to the patch in the future, both rewards should be presented, even if one is only presented as a secondary reward. There was a significant positive correlation between the sugar content and pollen production of flowers in this study, suggesting that these traits are not completely independent of each other. Nonetheless, a positive relationship between pollen and nectar production should not be prohibitive to breeding for more rewarding flowers to pollinators. Furthermore, this relationship is the opposite of that expected if there were a trade‐off in resource use within a flower within the range examined in this study, indicating that further increases in reward production may be possible.

One aspect of floral reward that has received less attention in previous studies is the force required for bees to access the reward (operative strength) of a flower. The operative strength we have measured here for *V. faba* (17 and 20 mN for lines with different sized flowers) is comparable to the 15–30 mN reported in alfalfa (Knapp & Teuber, 1994). An increase in the operative strength of flowers has the potential to decrease pollination rates by two mechanisms. Firstly, the operative strength will reduce the overall reward of the flower by increasing the energy required to obtain a reward from a flower. Secondly, it will exclude any pollinators too weak to manipulate the flower. If we assume that the flower opening behaves like a spring, with a spring constant of *k* = 20 mN/cm (the force required to move the wing petal ~1 cm and trip the flower), then the mean energy applied to the petal hinge (from 0 (0 mN) to 1 cm (20 mN) deflection) is 10 mN which is equivalent to 0.0001 J. The mechanical component of the energetic cost of opening a *V. faba* flower is therefore negligible (~0.001%) compared with the mean nectar reward of 0.6 mg sugar (equivalent to 9.8 J assuming the sugar is sucrose). In contrast, the operative strength of a flower could have much greater implications for the visitation rates of the flower by influencing flower accessibility. Opening *V. faba* flowers should be relatively easy for *Bombus* spp., which can exert over 200 mN of force. However, *Apis mellifera* is reported to be able to only exert ~26 mN of force on average (Córdoba & Cocucci, 2011), therefore weaker bees of this species and other smaller bee species such as *Eucera numida* (which pollinates *V. faba* in Spain) may have difficulty opening stronger than average flowers. Data from alfalfa suggest that flowers with a lower operative force are tripped more often and set more seed in the field, although honeybee visitation was not significantly different (Knapp & Teuber, 1990). This suggests that breeding for lower operative strengths can increase yield independently of pollinator visitation rates by increasing the proportion of flowers that are visited successfully. This mechanism of increasing pollinator visitation to flowers therefore warrants further investigation, particularly with respect to its relationship to flower size.

## 5 CONCLUSIONS

Overall, the results of this study suggest that there is clear genetic potential to alter the floral traits of *V. faba* flowers in a way that could enhance bee visitation and support pollinator populations. This could be achieved by breeding traits that are preferable to bees from landraces (locally adapted lines which have not undergone intensive selection by breeders) and lines no longer being commercially grown into current commercially grown varieties with preferable agronomic traits. Bees may then preferentially visit fields of optimized varieties over less rewarding flowers in the environment, based on location cues. Similar possibilities for nectar production have also recently been shown in two other pollinator‐dependent crops—sunflower and oilseed rape (Carruthers et al., 2017; Mallinger & Prasifka, 2017). We suggest for *V. faba* that this could be achieved most effectively by improving the pollen production and nectar sugar concentration of flowers (but not above 55% w/w), while maintaining an average volume of nectar in flowers. However, field trials are required to confirm this. This strategy should increase the number of legitimate visits to the crop by increasing the number of pollen foragers, while not deterring nectar foragers. These improvements to the nutritional quality of the crop may also have indirect benefits, by supporting local pollinator populations and enhancing yield in future years (Holzschuh, Dormann, Tscharntke, & Steffan‐Dewenter, 2013; Jauker, Peter, Wolters, & Diekötter, 2012). This would be especially true if used in combination with interventions such as wild‐flower strips to increase the amount of forage available when the crop is not flowering (Carvell et al., 2017; Pywell et al., 2015). Our measurement of the operative strength of flowers suggests that the importance of breeding for flowers that are easier to open will depend on the pollinator assemblage present. Where bumblebees are the main pollinator, breeding for a lower operative strength is unlikely to alter visitation rates. Future work is now needed to verify whether the genetic potential for breeding more attractive flowers, such as flowers with higher nectar concentrations, can translate into higher visitation rates and yield stability in the field. Interestingly, the lines in this study that are derivatives of recent commercial varieties (Table S1) tend to have higher nectar and pollen production compared with the land races in this study. This suggests that indirect selection has already improved the reward production of flowers to some extent and that targeted breeding has great potential to exploit this diversity further.

## DATA ACCESSIBILITY

Supporting data is available at https://doi.org/10.17863/cam.17142

## CONFLICT OF INTEREST

None declared.

## AUTHORS' CONTRIBUTIONS

B.J.G. and E.J.B. conceived the ideas and designed methodology; E.J.B. and J.G.P. collected the data; E.J.B. and J.G.P. analyzed the data; E.J.B. led the writing of the manuscript. All authors contributed critically to the drafts and gave final approval for publication.

## References

[ece33851-bib-0001] Aizen, M. A. , & Harder, L. D. (2009). The global stock of domesticated honey bees is growing slower than agricultural demand for pollination. Current Biology, 19, 915–918. https://doi.org/10.1016/j.cub.2009.03.071 19427214 10.1016/j.cub.2009.03.071

[ece33851-bib-0002] Ashman, T.‐L. (1994). A dynamic perspective on the physiological cost of reproduction in plants. The American Naturalist, 144, 300–316. https://doi.org/10.1086/285676

[ece33851-bib-0003] Bailes, E. J. , Ollerton, J. , Pattrick, J. G. , & Glover, B. J. (2015). How can an understanding of plant‐pollinator interactions contribute to global food security? Current Opinion in Plant Biology, 26, 72–79. https://doi.org/10.1016/j.pbi.2015.06.002 26116979 10.1016/j.pbi.2015.06.002

[ece33851-bib-0004] Bates, D. , Mächler, M. , Bolker, B. , & Walker, S. (2015). Fitting linear mixed‐effects models using lme4. Journal of Statistical Software, 67, 1–48.

[ece33851-bib-0005] Bishop, J. , Jones, H. E. , Lukac, M. , & Potts, S. G. (2016). Insect pollination reduces yield loss following heat stress in faba bean (*Vicia faba* L.). Agriculture, Ecosystems & Environment, 220, 89–96. https://doi.org/10.1016/j.agee.2015.12.007 10.1016/j.agee.2015.12.007 PMC4767028 26989276

[ece33851-bib-0006] Brunet, J. , Thairu, M. W. , Henss, J. M. , Link, R. I. , & Kluever, J. A. (2015). The effects of flower, floral display, and reward sizes on bumblebee foraging behavior when pollen is the reward and plants are dichogamous. International Journal of Plant Sciences, 176, 811–819. https://doi.org/10.1086/683339

[ece33851-bib-0007] Carré, S. , Badenhauser, I. , Taséi, J. N. , Le Guen, J. , & Mesquida, J. (1994). Pollen deposition by *Bombus terrestris* L, between male‐fertile and male‐sterile plants in *Vicia faba* L. Apidologie, 25, 338–349. https://doi.org/10.1051/apido:19940310

[ece33851-bib-0008] Carruthers, J. M. , Cook, S. M. , Wright, G. A. , Osborne, J. L. , Clark, S. J. , Swain, J. L. , & Haughton, A. J. (2017). Oilseed rape (*Brassica napus*) as a resource for farmland insect pollinators: Quantifying floral traits in conventional varieties and breeding systems. GCB Bioenergy, 9, 1370–1379. https://doi.org/10.1111/gcbb.12438 28781612 10.1111/gcbb.12438 PMC5518758

[ece33851-bib-0009] Carvell, C. , Bourke, A. F. G. , Dreier, S. , Freeman, S. N. , Hulmes, S. , Jordan, W. , … Heard, M. S. (2017). Bumblebee family lineage survival is enhanced in high quality landscapes. Nature, 543, 547–549. https://doi.org/10.1038/nature21709 28297711 10.1038/nature21709

[ece33851-bib-0010] Cnaani, J. , Thomson, J. D. , & Papaj, D. R. (2006). Flower choice and learning in foraging bumblebees: Effects of variation in nectar volume and concentration. Ethology, 112, 278–285. https://doi.org/10.1111/j.1439-0310.2006.01174.x

[ece33851-bib-0011] Cook, S. M. , Awmack, C. S. , Murray, D. A. , & Williams, I. H. (2003). Are honey bees' foraging preferences affected by pollen amino acid composition? Ecological Entomology, 28, 622–627. https://doi.org/10.1046/j.1365-2311.2003.00548.x

[ece33851-bib-0012] Córdoba, S. A. , Benitez‐Vieyra, S. , & Cocucci, A. A. (2015). Functional modularity in a forcible flower mechanism: Relationships among morphology, biomechanical features and fitness. Evolutionary Ecology, 29, 719–732. https://doi.org/10.1007/s10682-015-9783-6

[ece33851-bib-0013] Córdoba, S. A. , & Cocucci, A. A. (2011). Flower power: Its association with bee power and floral functional morphology in papilionate legumes. Annals of Botany, 108, 919–931. https://doi.org/10.1093/aob/mcr196 21821623 10.1093/aob/mcr196 PMC3177674

[ece33851-bib-0014] Cresswell, J. E. (1999). The influence of nectar secretion rates on the responses of bumblebees (*Bombus* spp.) to previously visited flowers. Journal of Ecology, 87, 670–677. https://doi.org/10.1046/j.1365-2745.1999.00385.x

[ece33851-bib-0015] Cunningham, S. A. , & Le Feuvre, D. (2013). Significant yield benefits from honeybee pollination of faba bean (*Vicia faba*) assessed at field scale. Field Crops Research, 149, 269–275. https://doi.org/10.1016/j.fcr.2013.05.019

[ece33851-bib-0101] FAO ‐ Food and Agriculture Organization of the United Nations , (2016). FAOSTAT (Database). Retrieved from http://www.fao.org/faostat/en/#data/QC

[ece33851-bib-0016] Garibaldi, L. A. , Carvalheiro, L. G. , Vaissière, B. E. , Gemmill‐herren, B. , Hipólito, J. , Freitas, B. M. , … An, J. (2016). Mutually beneficial pollinator diversity and crop yield outcomes in small and large farms. Science, 351, 388–391. https://doi.org/10.1126/science.aac7287 26798016 10.1126/science.aac7287

[ece33851-bib-0017] Garratt, M. P. D. , Breeze, T. D. , Jenner, N. , Polce, C. , Biesmeijer, J. C. , & Potts, S. G. (2014). Avoiding a bad apple: insect pollination enhances fruit quality and economic value. Agriculture, Ecosystems & Environment, 184, 34–40. https://doi.org/10.1016/j.agee.2013.10.032 10.1016/j.agee.2013.10.032 PMC3990452 24748698

[ece33851-bib-0018] Garratt, M. P. D. , Coston, D. J. , Truslove, C. L. , Lappage, M. G. , Polce, C. , Dean, R. , … Potts, S. G. (2014). The identity of crop pollinators helps target conservation for improved ecosystem services. Biological Conservation, 169, 128–135. https://doi.org/10.1016/j.biocon.2013.11.001 24696525 10.1016/j.biocon.2013.11.001 PMC3969722

[ece33851-bib-0019] Groen, S. C. , Jiang, S. , Murphy, A. M. , Cunniffe, N. J. , Westwood, J. H. , Davey, M. P. , … Robinson, S. I. (2016). Virus infection of plants alters pollinator preference: A payback for susceptible hosts? PLoS Pathogens, 12, 1–28. 10.1371/journal.ppat.1005790 PMC4981420 27513727

[ece33851-bib-0102] Hagbery, J. , & Nieh, J. (2012a). Individual lifetime pollen and nectar foraging preferences in bumble bees. Naturwissenschaften, 99, 821–832. https://doi.org/10.1007/s00114-012-0964-7 22965265 10.1007/s00114-012-0964-7

[ece33851-bib-0020] Harder, L. D. (1986). Effects of nectar concentration and flower depth on flower handling efficiency of bumble bees. Oecologia, 69, 309–315. https://doi.org/10.1007/BF00377639 28311376 10.1007/BF00377639

[ece33851-bib-0021] Holzschuh, A. , Dormann, C. F. , Tscharntke, T. , & Steffan‐Dewenter, I. (2013). Mass‐flowering crops enhance wild bee abundance. Oecologia, 172, 477–484. https://doi.org/10.1007/s00442-012-2515-5 23114428 10.1007/s00442-012-2515-5 PMC3655217

[ece33851-bib-0022] Jauker, F. , Peter, F. , Wolters, V. , & Diekötter, T. (2012). Early reproductive benefits of mass‐flowering crops to the solitary bee *Osmia rufa* outbalance post‐flowering disadvantages. Basic and Applied Ecology, 13, 268–276. https://doi.org/10.1016/j.baae.2012.03.010

[ece33851-bib-0023] Kambal, A. E. , Bond, D. A. , & Toynbee‐Clarke, G. (1976). A study on the pollination mechanism in field beans (*Vicia faba* L.). The Journal of Agricultural Science, 87, 519–526. https://doi.org/10.1017/S0021859600033128

[ece33851-bib-0024] Klatt, B. K. , Holzschuh, A. , Westphal, C. , Clough, Y. , Smit, I. , Pawelzik, E. , & Tscharntke, T. (2014). Bee pollination improves crop quality, shelf life and commercial value. Proceedings of the Royal Society B: Biological Sciences, 281, 20132440. 24307669 10.1098/rspb.2013.2440 PMC3866401

[ece33851-bib-0025] Klein, A. M. , Vaissière, B. E. , Cane, J. H. , Steffan‐Dewenter, I. , Cunningham, S. A. , Kremen, C. , & Tscharntke, T. (2007). Importance of pollinators in changing landscapes for world crops. Proceedings of the Royal Society B: Biological Sciences, 274, 303–313. https://doi.org/10.1098/rspb.2006.3721 17164193 10.1098/rspb.2006.3721 PMC1702377

[ece33851-bib-0026] Knapp, E. E. , & Teuber, L. R. (1990). Environmental factors and plant phenotype affect alfalfa floret. Crop Science, 30, 270–275. https://doi.org/10.2135/cropsci1990.0011183X003000020006x

[ece33851-bib-0027] Knapp, E. E. , & Teuber, L. R. (1994). Selection progress for ease of floret tripping in alfalfa. Crop Science, 34, 323–326. https://doi.org/10.2135/cropsci1994.0011183X003400020001x

[ece33851-bib-0028] Kobayashi, K. , Tsukamoto, S. , Tanaka, A. , Niikura, S. , & Ohsawa, R. (2010). Selective flower visitation behavior by pollinators in a radish F1 seed production field. Breeding Science, 60, 203–211. https://doi.org/10.1270/jsbbs.60.203

[ece33851-bib-0103] Konzmann, S. , & Lunau, K. (2014). Divergent rules for pollen and nectar foraging bumblebees ‐ A laboratory study with artificial flowers offering diluted nectar substitute and pollen surrogate. PLoS ONE, 9, e91900 https://doi.org/10.1371/journal.pone.0091900 24637406 10.1371/journal.pone.0091900 PMC3956814

[ece33851-bib-0029] Mallinger, R. E. , & Prasifka, J. R. (2017). Bee visitation rates to cultivated sunflowers increase with the amount and accessibility of nectar sugars. Journal of Applied Entomology, 141, 1–13. https://doi.org/10.1111/jen.12375

[ece33851-bib-0030] Mommaerts, V. , Wäckers, F. , & Smagghe, G. (2013). Assessment of gustatory responses to different sugars in harnessed and free‐moving bumblebee workers (*Bombus terrestris*). Chemical Senses, 38, 399–407. https://doi.org/10.1093/chemse/bjt014 23599218 10.1093/chemse/bjt014

[ece33851-bib-0031] Nadal, S. , Suso, M. J. , & Moreno, M. T. (2003). Management of *Vicia faba* genetic resources: Changes associated to the selfing process in the major, equina and minor groups. Genetic Resources and Crop Evolution, 50, 183–192. https://doi.org/10.1023/A:1022944017530

[ece33851-bib-0032] Nayak, G. K. , Roberts, S. P. M. , Garratt, M. P. D. , Breeze, T. D. , Tscheulin, T. , Harrison‐Cripps, J. , … Potts, S. G. (2015). Interactive effect of floral abundance and semi‐natural habitats on pollinators in field beans (*Vicia faba*). Agriculture, Ecosystems & Environment, 199, 58–66. https://doi.org/10.1016/j.agee.2014.08.016

[ece33851-bib-0033] Ollerton, J. , Killick, A. , Lamborn, E. , Watts, S. , & Whiston, M. (2007). Multiple meanings and modes: On the many ways to be a generalist Flower. Taxon, 56, 717–728. https://doi.org/10.2307/25065856

[ece33851-bib-0034] Osborne, J. L. , Awmack, C. S. , Clark, S. J. , Williams, I. H. , & Mills, V. C. (1997). Nectar and flower production in *Vicia faba* L (field bean) at ambient and elevated carbon dioxide. Apidologie, 28, 43–55. https://doi.org/10.1051/apido:19970105

[ece33851-bib-0035] Palmer, R. G. , Perez, P. T. , Ortiz‐Perez, E. , Maalouf, F. , & Suso, M. J. (2009). The role of crop‐pollinator relationships in breeding for pollinator‐friendly legumes: From a breeding perspective. Euphytica, 170, 35–52. https://doi.org/10.1007/s10681-009-9953-0

[ece33851-bib-0036] Pierre, J. , Le Guen, J. , Pham‐Delègue, M. H. , Mesquida, J. , Marilleau, R. , & Morin, G. (1996). Comparative study of nectar secretion and attractivity to bees of two lines of spring‐type faba bean (*Vicia faba* L var equina Steudel). Apidologie, 27, 66–75.

[ece33851-bib-0037] Pinheiro, J. , Bates, D. , DebRoy, S. , & Sarkar, D. and R Core Team (2017). nlme: Linear and Nonlinear Mixed Effects Models. R package version 3.1‐131.

[ece33851-bib-0038] Pywell, R. F. , Heard, M. S. , Woodcock, B. A. , Hinsley, S. , Ridding, L. , Nowakowski, M. , & Bullock, J. M. (2015). Wild‐life friendly farming increases crop yield: Evidence for ecological intensification. Proceedings of the Royal Society of London. Series B, Biological Sciences, 282, 20151740 https://doi.org/10.1098/rspb.2015.1740 26423846 10.1098/rspb.2015.1740 PMC4614778

[ece33851-bib-0039] R Core Team (2017). R: A language and environment for statistical computing.

[ece33851-bib-0040] Richards, R. A. (2000). Selectable traits to increase crop photosynthesis and yield of grain crops. Journal of Experimental Botany, 51, 447–458. https://doi.org/10.1093/jexbot/51.suppl_1.447 10938853 10.1093/jexbot/51.suppl_1.447

[ece33851-bib-0041] Robertson, A. W. , Mountjoy, C. , Faulkner, B. E. , Roberts, M. V. , & Macnair, M. R. (1999). Bumble bee selection of *Mimulus guttatus* flowers: The effects of pollen quality and reward depletion. Ecology, 80, 2594–2606. https://doi.org/10.1890/0012-9658(1999)080[2594:BBSOMG]2.0.CO;2

[ece33851-bib-0042] Somme, L. , Vanderplanck, M. , Michez, D. , Lombaerde, I. , Moerman, R. , Wathelet, B. , … Jacquemart, A. L. (2015). Pollen and nectar quality drive the major and minor floral choices of bumble bees. Apidologie, 46, 92–106. https://doi.org/10.1007/s13592-014-0307-0

[ece33851-bib-0043] Suso, M. J. , & del Río, R. (2015). A crop–pollinator inter‐play approach to assessing seed production patterns in faba bean under two pollination environments. Euphytica, 201, 231–251. https://doi.org/10.1007/s10681-014-1200-7

[ece33851-bib-0044] Suso, M. J. , Harder, L. D. , Moreno, M. T. , & Maalouf, F. (2005). New strategies for increasing heterozygosity in crops: *Vicia faba* mating system as a study case. Euphytica, 143, 51–65. https://doi.org/10.1007/s10681-005-2526-y

[ece33851-bib-0045] Suso, M. J. , & Maalouf, F. (2010). Direct and correlated responses to upward and downward selection for outcrossing in *Vicia faba* . Field Crops Research, 116, 116–126. https://doi.org/10.1016/j.fcr.2009.12.001

[ece33851-bib-0046] Suso, M. J. , Nadal, S. , Roman, B. , & Gilsanz, S. (2008). *Vicia faba* germplasm multiplication – floral traits associated with pollen‐mediated gene flow under diverse between‐plot isolation strategies. Annals of Applied Biology, 152, 201–208. https://doi.org/10.1111/j.1744-7348.2007.00205.x

[ece33851-bib-0047] Tester, M. , & Langridge, P. (2010). Breeding technologies to increase crop production in a changing world. Science, 327, 818–822. https://doi.org/10.1126/science.1183700 20150489 10.1126/science.1183700

[ece33851-bib-0048] Thomson, J. D. , Ogilvie, J. E. , Makino, T. T. , Arisz, A. , Raju, S. , Guo, M. , & Tan, R. (2012). Estimating pollination success with novel artificial flowers: Effects of nectar concentration. Journal of Pollination Ecology, 9, 108–114.

[ece33851-bib-0049] Vaudo, A. D. , Patch, H. M. , Mortensen, D. A. , Tooker, J. F. , & Grozinger, C. M. (2016). Macronutrient ratios in pollen shape bumble bee *(Bombus impatiens*) foraging strategies and floral preferences. Proceedings of the National Academy of Sciences, 113, E4035–E4042. https://doi.org/10.1073/pnas.1606101113 10.1073/pnas.1606101113 PMC4948365 27357683

[ece33851-bib-0050] Waddington, K. D. , & Gottlieb, N. (1990). Actual vs perceived profitability: A study of floral choice of honey bees. Journal of Insect Behavior, 3, 429–441. https://doi.org/10.1007/BF01052010

[ece33851-bib-0051] Waller, G. D. (1972). Evaluating responses of honey bees to sugar solutions using an artificial‐flower feeder. Annals of the Entomological Society of America, 65, 857–862. https://doi.org/10.1093/aesa/65.4.857

[ece33851-bib-0052] Whitney, H. , Dyer, A. G. , Chittka, L. , Rands, S. , & Glover, B. J. (2008). The interaction of temperature and sucrose concentration on foraging preferences in bumblebees. Naturwissenschaften, 95, 845–850. https://doi.org/10.1007/s00114-008-0393-9 18523748 10.1007/s00114-008-0393-9

[ece33851-bib-0053] Woodrow, A. W. (1968). Some factors affecting selection of sucrose solutions by foraging honey bees. American Bee Journal, 108, 313–315.

