# Supplementary material for: An analysis of the energetic reward offered by field bean (Vicia faba) flowers: Nectar, pollen, and operative force
Source: Ecol Evol. 2018 Feb 18;8(6):3161–71. doi: 10.1002/ece3.3851 (PMC5869266; doi:10.1002/ece3.3851)
Supplement: Supplementary file 1 [file ECE3-8-3161-s001.docx]

**Supplementary information:**

*Supplementary methods*

**Table S1 -** The seed source for the lines used in this study. Information supplied by the National Institute of Agricultural Botany. The floral traits quantified for each line are given.

| **Line** | **Seed source** | **Donor ref and other names** | **Origin** | **Floral traits measured** | | |
| --- | --- | --- | --- | --- | --- | --- |
|  |  |  |  | **Nectar production** | **Pollen production** | **Opening force** |
| NV020 | ICARDA | ig11290 | landrace | Y | Y | - |
| NV027 | ICARDA | ig11312 | landrace | Y | Y | - |
| NV079 | ICARDA | ig11687 | landrace | Y | Y | - |
| NV082 | ICARDA | ig11695 | landrace | Y | Y | - |
| NV100 | ICARDA | ig11749 | landrace | Y | Y | - |
| NV129 | ICARDA | ig12137 | landrace | Y | Y | - |
| NV155 | ICARDA | ig12684 | landrace | Y | Y | - |
| NV175 | ICARDA | ig13004 | landrace | Y | - | - |
| NV293 | ICARDA | ig70726 | landrace | Y | Y | - |
| NV490 | ICARDA | ig124213 | landrace | Y | Y | - |
| NV574 | ICARDA | ig130638 | landrace | Y | - | - |
| NV604 | JIC | V185, Borington Bulk | landrace | Y | Y | - |
| NV619 | NICK-ADV | NA12,  LAN08935 | landrace | Y | Y | - |
| NV620 | CSIC | CSIC,  Vf172 | landrace | Y | Y | - |
| NV626 | CBP-T | KWS6,  NPZ 7-7301 | landrace | Y | Y | - |
| NV639 | GOTTINGEN | 70176/70175, Hedin | commercial variety | Y | Y | - |
| NV640 | NIAB | Maris Bead | commercial variety | Y | Y | - |
| NV641 | NIAB | Fuego | commercial variety | Y | Y | Y |
| NV643 | POL | Albus | commercial variety | Y | Y | - |
| NV644 | POL | Kasztelan | commercial variety | Y | Y | - |
| NV648 | ICARDA | ig101769, BPL10 | landrace | Y | Y | - |
| NV649 | ICARDA | ig101770, BPL11 | landrace | Y | Y | - |
| NV650 | ICARDA | ig101771, BPL12 | landrace | Y | Y | - |
| NV653 | ICARDA | ig101786, BPL27 | landrace | Y | Y | - |
| NV658 | GOTTINGEN | CGN07715 cf-3 (60354-9), closed flower mutant | landrace | Y | Y | - |
| NV671 | NIAB | Atlas | commercial variety | Y | Y | - |
| NV673 | NIAB | Fury | commercial variety | Y | Y | - |
| NV675 | NIAB | Pyramid | commercial variety | Y | Y | - |
| NV676 | NIAB | Tattoo | commercial variety | Y | Y | Y |
| NV706 | Thompson & Morgan | Broad Bean Crimson Flowered | horticultural variety | Y | Y | - |

**Pollen content**

The anthers from 10 buds of the same plant were collected in a 15 ml falcon tube and allowed to dehisce. The pollen was then re-suspended in 5 ml of a 1:1 0.1 % Tween 80 v/v: 0.1 % w/v agar solution by vortexing at high speed for 60 seconds. The suspension was then filtered through a 355 µm mesh and 20 subsamples of the solution were counted using a haemocytometer slide (AC1000, Hawksey, England). The total number of pollen grains in each flower was estimated following equation 1. The pollen production of five to eight plants was estimated for a total of 28 lines (specific sample sizes in Table 1). . Pollen production was not scored for lines NV175 and NV574. The distribution of samples across the months of sampling are given in Figure S1.

**Equation 1**:

Pollen grains per flower = 500 (average number of pollen grains per subsample/0.9)

where 500 is the volume (µL) each flower was re-suspended in and 0.9 is the volume (µl) of each subsample counted.


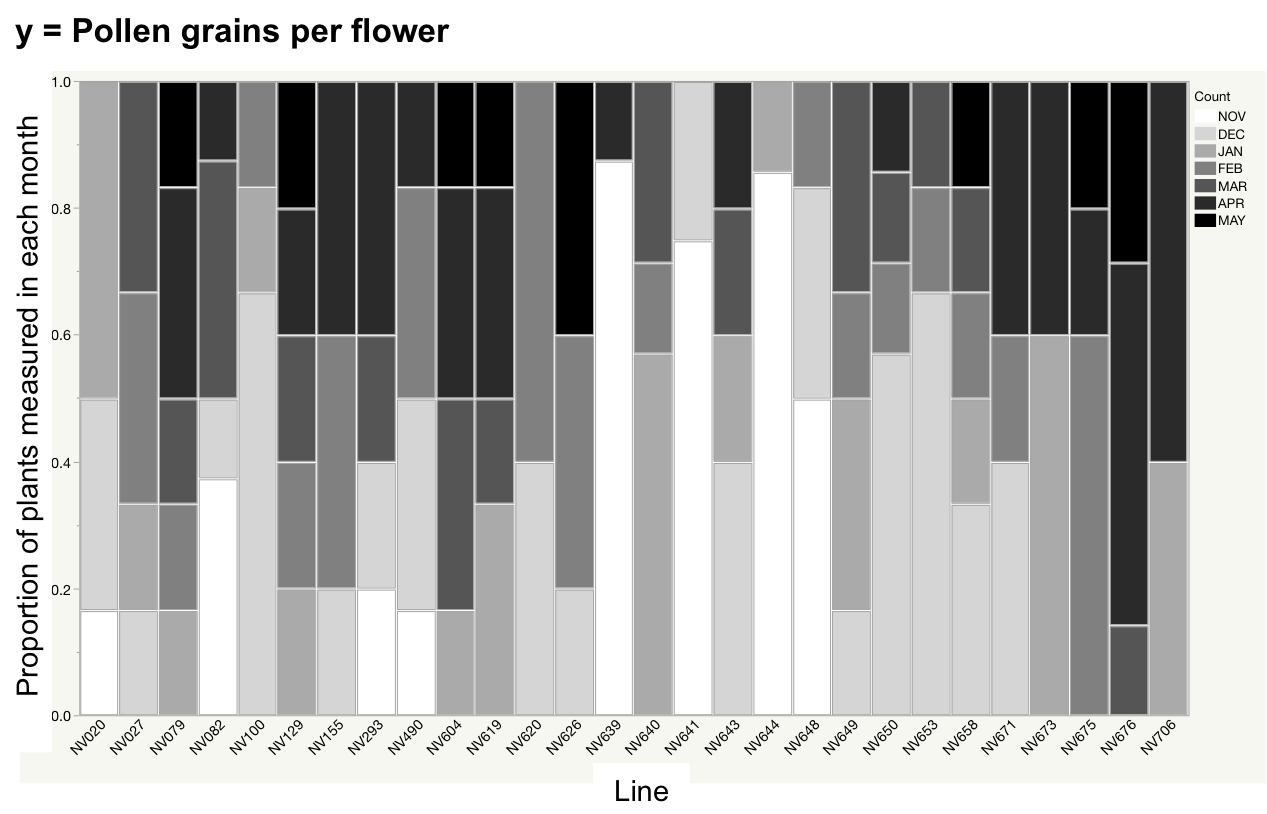


**Figure S1** – The distribution of data between months within each line for our model of the number of pollen grains produced by a flower. Shades of grey indicate the month flowers were measured in (see key above) and the height of the bar represents the proportion of total data points from that line that were measured in a particular month.

**Nectar production**

To estimate the nectar production of flowers, open flowers of stage four to five, as described by Osborne et al. (1997), were removed in a random order between 10 am and noon from plants that had been flowering for between one and three weeks. These restrictions on the collection time of flowers allowed us to control for potential variations in nectar production with time of day and plant age. Immediately following the collection of stage 4 or 5 flowers, the petals and anthers of each flower were removed to expose the nectaries at the base of the reproductive complex. Excess nectar that had collected at the base of the standard was removed using 0.5, 1, 2 or 5 μl calibrated micro-capillaries (Drummond Scientific, US). The reproductive complex was then sealed in a 0.5 ml microcentrifuge tube with 3 holes pierced at its base, and this tube placed within a 1.5 ml tube. The nectar from each flower was then collected by centrifugation of the 1.5 ml tubes at 13,000 rpm for one minute. The reproductive complex was then dislodged from the inner tube and spun again for a second minute at 13,000 rpm to collect any residual nectar.

The volume of nectar collected by centrifugation was estimated by dividing the change in mass in the outer collection tube before and after nectar collection by the density of the sample. The density of the sample was estimated using the sugar concentration of that sample following the formula of Prŷs-Jones and Corbet (2011; see below), assuming all sugars are sucrose (the predominant sugar contained in *V. faba* nectar (Pierre et al., 1996)). This estimated volume was added to the volume of excess nectar collected using micro-capillaries.

$$density \left( mg/\mu L \right)= 0.0037291C+0.0000178\boldsymbol{C}^{2}+0.9988603$$

where **C** is the measured concentration of the nectar (measured using a refractometer – models Bellingham + Stanley, Eclipse 45-03 or Bellingham + Stanley, Eclipse 45-82) in % sugar w/w (ie. 55 for 55% w/w sugar concentration). Where the volume of nectar collected was inadequate to measure its sugar concentration, an estimation of sugar concentration based on the grand mean concentration of other flowers within that plant or line was used to determine nectar volume.

The total mass of sugar produced per flower (mg) was calculated by multiplying the density (mg/µl) of solution (above) by the total estimated volume (µl) and proportion of that weight attributable to sugar (ie. 0.55 for 55% w/w sugar concentration).

It was not possible to measure the concentration for all of the nectar collected due to low volumes produced by many flowers. Overall, the concentration was measured for 74% of flowers, but this was distributed unevenly between lines. The concentration and sugar content analyses were not run on lines NV658 and NV155 because they had concentration data for less than 25% of flowers (22% and 1% respectively). The level of replication within each line is given in Table 1.

Line NV641 was measured throughout the study period, months other lines were grown are indicated in Figures S2 and S3


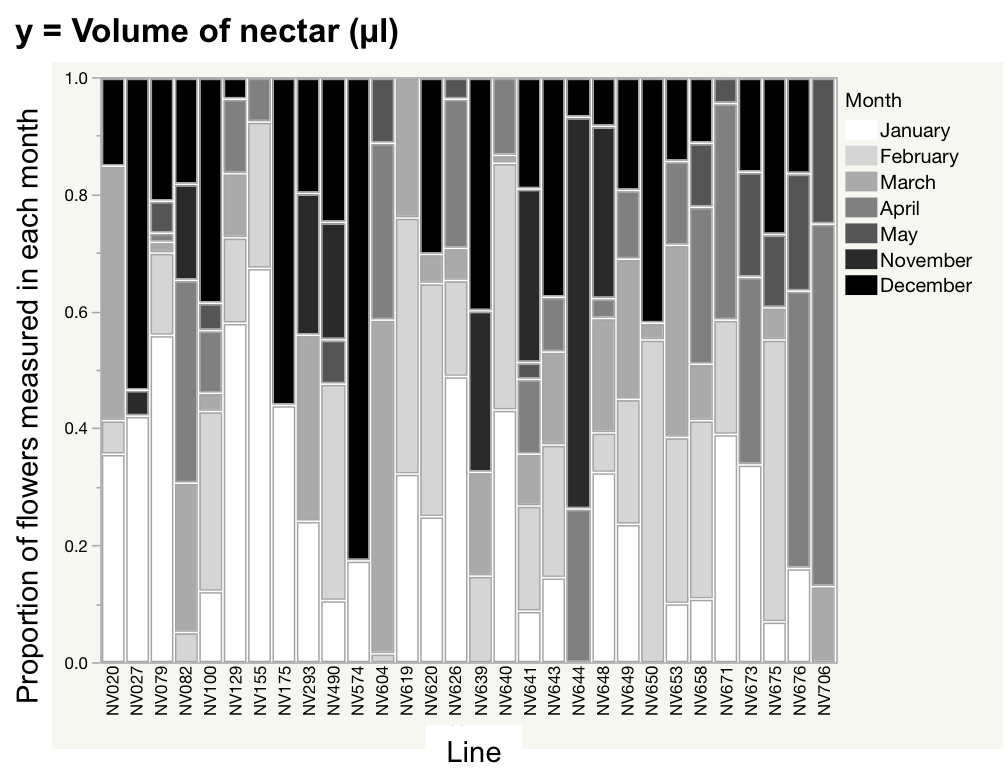


**Figure S2 –** The distribution of data between months within each line for our model of the volume of nectar produced by a flower (µl). The analyses of sugar mass in the main text also used these flowers, but excluded data points from NV155 & NV658. Shades of grey indicate the month the flowers were measured in (see key above) and the height of the bar represents the proportion of total data points from that line that were measured in a particular month.


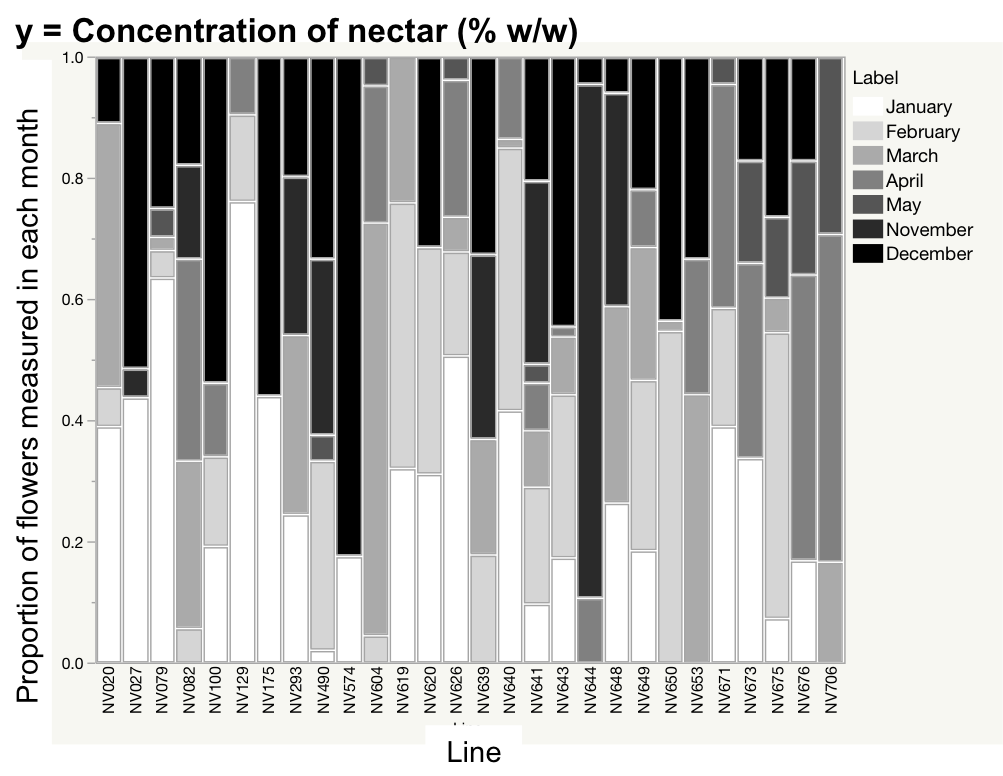


**Figure S3** – The distribution of data between months within each line for our model of the concentration of nectar produced by a flower (%w/w). The analyses of sugar without estimates used these flowers. Shades of grey indicate the month flowers were measured in (see key above) and the height of the bar represents the proportion of total data points from that line that were measured in a particular month.

**The operative strength of a flower**

A dynamometer (Model 20010 (10g) PESODA, Switzerland) with a measurement range of 0.1 – 10g was held vertically on a metal frame (see Fig S4). Open flowers (stage four - five; Osborne et al., 1997) were suspended from the dynamometer using a metal clip attached to the base of the calyx. The dynamometer was tared to account for the weight of both the flower and the clip suspending it. Following this a blunt crocodile clip was attached to the flower’s right-hand wing petal at its center, where a pollinator will usually push against to gain access to the flower. This differs from the method of Cordoba & Cocucci (2011) as when their method was attempted on *V. faba*, the wing petals simply bent down and the flower did not trip. The clip was steadily lowered by turning the screw until the flower was tripped. At this point the force measured by the dynamometer was recorded. Tripping was defined as when the stigmatic surface was visible emerging from the keel petals. The measurement of operative force (g) was then converted into mN. Plants grown for these measurements were maintained in a growth cabinet at light levels of 150 μmol for a 16 h day, the temperature was maintained at 20 °C, and humidity 60 %. The distribution of data between dates of measurement is given in Fig S5.


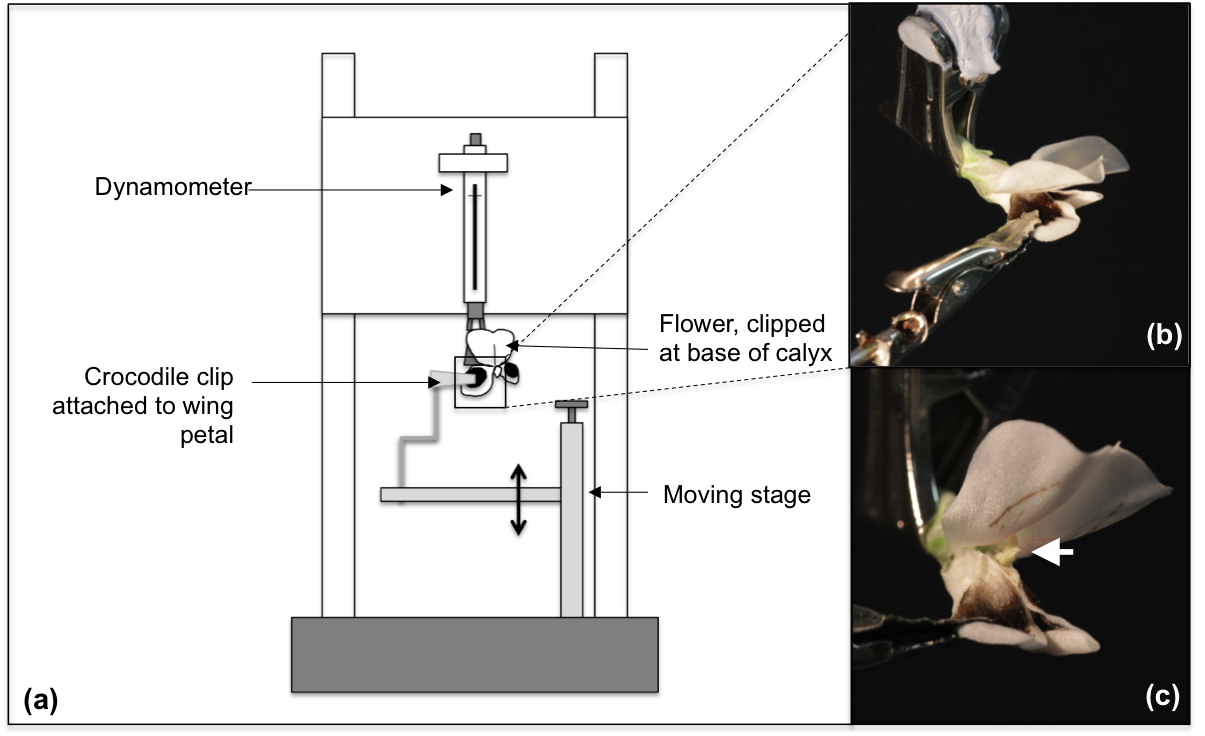


**Figure S4.** Apparatus setup to measure the force required to trip a flower of *V. faba*. (a & b) A freshly abscised stage 5 flower was secured to a dynamometer by the base of the calyx, the wing petal was then connected to a moving stage using a crocodile clip. (c) The stage was moved downwards until the stigmatic surface was visible (white arrow).

**
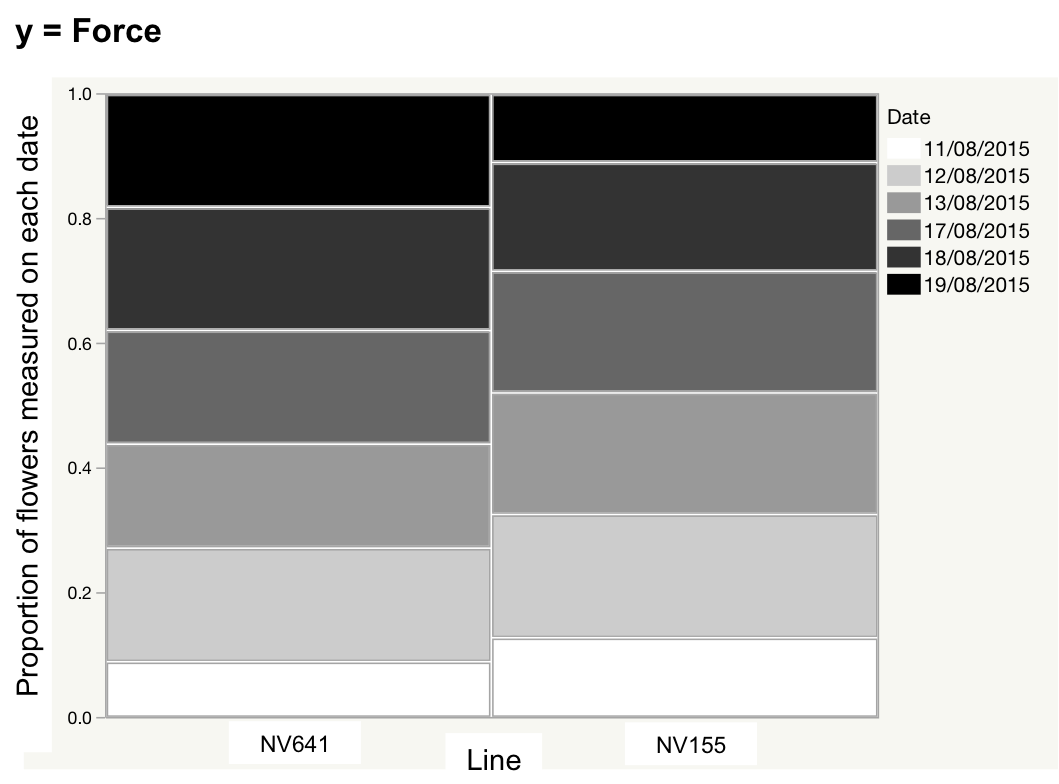
**

**Figure S5** – The distribution of data between days within each line for our model of the operative strength of a flower (mN). Shades of grey indicate the date flowers were measured on (see key above) and the height of the bar represents the proportion of total data points from that line that were measured on a particular date.

**Bee behavioural experiments:**

To determine whether bumblebee foragers (*Bombus terrestris audax* (supplied by Agralan, UK)) have a preference between different sugar concentrations, pair-wise comparisons were made, using the combinations 40 % w/w and 55 % w/w sugar solution, and 55 % w/w and 68 % w/w sugar solution. These were chosen to represent roughly the average sugar concentration of nectar across our variation panel, the highest average sugar concentration of nectar within a line, and the highest plant average sugar concentration in our dataset.

Experiments were carried out in a 0.3 x 0.75 x 1.12 m plywood flight arena with a clear UV-transparent Pexiglass lid. Colonies were fed *ad libitum* with ~30 % w/w sugar solution and pollen.

*Experimental set-up*

For each pair-wise comparison each sugar concentration was paired with a yellow or white coloured artificial disk. These disks were 4 cm in diameter and were produced by mixing epoxy-resin (ITW Devcon, USA) with pigment (Cornelissen & Son, London UK). These disks were widely separated in bee colour space when their location was calculated according to Chittka (1992) and therefore were an easy colour cue for the foragers to learn (Fig S2; Dyer and Chittka, 2004). During all experiments the disks were rewarded with 5 µl of sugar solution. Disks were placed on a 6 cm high tower to encourage flight between disks, there was a minimum of 12 cm between disk edges in the flight arena.


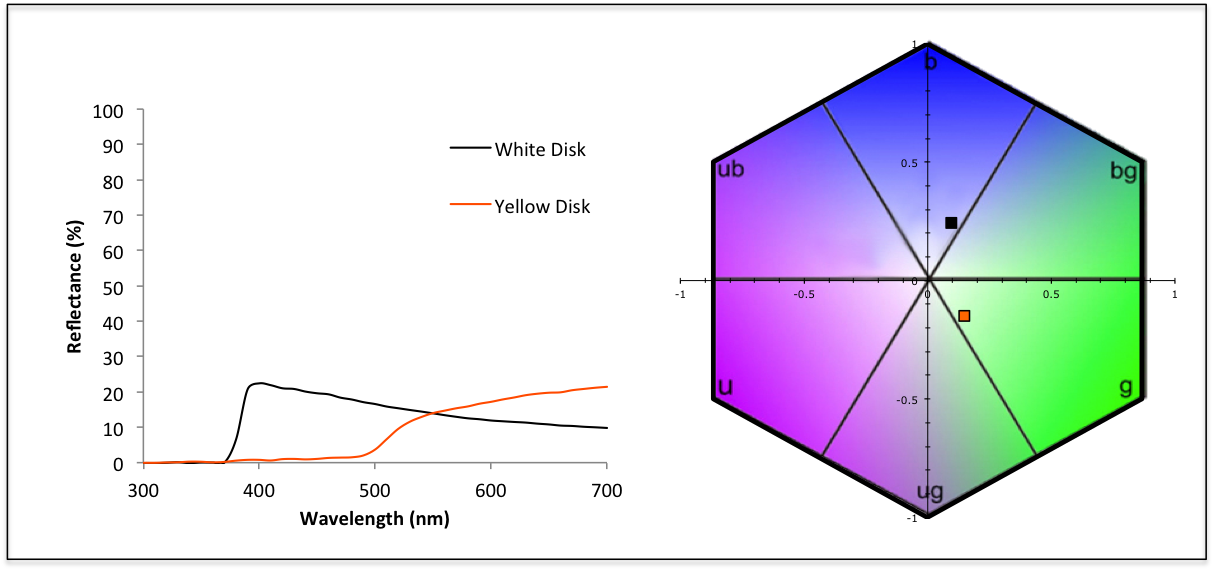


**Figure S2:** The colour of artificial disks used for bee behavioural experiments in bee colour space. (Left) The reflectance spectra of yellow (orange line) and white (black line) artificial disks are different in the range visible to bees. (Right) White (Black square) and yellow (Orange square) disks are widely separated in bee colour space and should be easily distinguishable by a bee.

*Training*

Before the experiment, each individual forager (marked with a unique colour code) was subjected to a training foraging bout to familiarize them with the foraging setup. This involved guiding the forager to the coloured disk with the lower sugar concentration using a Pasteur pipette containing sugar solution. This forager was then observed until it had visited the remaining coloured disk containing the higher concentration of sugar solution. Once this visit had been completed, the forager was allowed to return to the colony. For the second experiment (55% vs 68% comparison), the training phase was extended so that each forager was forced to visit four disks of each colour, which were presented in pairs, one of each colour.

*Experimental setup*

Following the training phase, the learning experiment to determine the preference of foragers for a particular sugar concentration was carried out as follows. Foragers were allowed into the arena containing an array of four yellow and four white disks. One hundred sequential choices of a forager were recorded, where a choice was defined as when the forager fed from an artificial flower. As a forager depleted the reward of each disk, it was replaced with a fresh disk with sugar reward in a new location in the arena. Between foraging bouts the arena floor was cleaned with 20 % ethanol to remove scent marks, as were disks before re-use. The choices of 10 foragers were recorded. To control for colour preferences, five foragers were assigned the high sugar concentration to the white disks, and low sugar concentration to the yellow disks and five foragers assigned the low sugar concentration to white disks and high sugar concentration to yellow disks.

*Supplementary results*

**Analyses of sugar production of flowers:**

For flowers where nectar volume was very small, so that direct concentration measurements were not possible, we estimated the concentration using the line mean. To investigate the effect of using these estimated values in our models, we analysed the sugar production of flower data both with and without these estimated values included.

When including estimated sugar concentrations the total sugar (mg sucrose equivalents) produced per flower ranged from less than 0.1 mg/flower in line NV620 (0.0 [0.0,0.1]) and NV129 (0.0 [0.0,0.1]) to a maximum of 1.7 [1.6,1.8] mg/flower in line NV619 and mean of 0.6 mg/flower.  **Line** (likelihood ratio = 334, p < 0.0001) and **Month** (likelihood ratio = 63) p < 0.0001) were both significant predictors of the sugar production of a flower. For the full model AIC = -969, which increased to -689 and -918 when the factors **Line** and **Month** were removed respectively.

When excluding estimated sugar concentrations the total sugar (mg sucrose equivalents) produced per flower range from 0.1 mg/flower in line NV620 (0.1 [0.1,0.2]) and NV129 (0.1 [0.1,0.2]) to a maximum of 1.7 [1.6,1.8] mg/flower in line NV619 and mean of 0.7 mg/flower. .**Line** (likelihood ratio = 320, p < 0.0001) and **Month** (likelihood ratio = 53, p < 0.0001) were both significant predictors of the sugar production of a flower. For the full model AIC = -936, which increased to -669 and -895 when the factors **Line** and **Month** were removed respectively.

Quantitatively there is very little difference in the estimated LS means for the lines between these models, with the means very slightly higher in their sugar production when considering only those flowers for which sugar concentration has been recorded. The back transformed means from the two models have a high correlation coefficient (R^2^ = 0.994).

Whilst there is very little difference between these two models, we feel that the former model containing all data will be a more accurate representation of the true sugar content of flowers within a line because this analysis does not bias the data to flowers where there were large enough volumes of nectar to quantify sugar concentration.

**References:**

Chittka, L. 1992. The colour hexagon: A chromaticity diagram based on photoreceptor excitations as a generalized representation of colour opponency. *Journal of Comparative Physiology A* 170: 533–543.

Córdoba, S.A., and A.A. Cocucci. 2011. Flower power: its association with bee power and floral functional morphology in papilionate legumes. *Annals of Botany* 108: 919–931.

Dyer, A.G., and L. Chittka. 2004. Fine colour discrimination requires differential conditioning in bumblebees. *Naturwissenschaften* 91: 224–227.

Osborne, J.L., C.S. Awmack, S.J. Clark, I.H. Williams, and V.C. Mills. 1997. Nectar and flower production in Vicia faba L (field bean) at ambient and elevated carbon dioxide. *Apidologie* 28: 43–55.

Pierre, J., J. Le Guen, M.H. Pham-Delègue, J. Mesquida, R. Marilleau, and G. Morin. 1996. Comparative study of nectar secretion and attractivity to bees of two lines of spring-type faba bean ( Vicia faba L var equina Steudel ). *Apidologie* 27: 66–75.

Prŷs-Jones, O., and S. Corbet. 2011. Bumblebees. Pelagic Publishing, Exeter.
